# Fluorescent Duplex Allele-Specific PCR and Amplicon Melting for Rapid Homogeneous mtDNA Haplogroup H Screening and Sensitive Mixture Detection

**DOI:** 10.1371/journal.pone.0008374

**Published:** 2009-12-18

**Authors:** Harald Niederstätter, Walther Parson

**Affiliations:** Institute of Legal Medicine, Innsbruck Medical University, Innsbruck, Austria; Erasmus University Medical Center, Netherlands

## Abstract

**Background:**

For large scale studies aiming at a better understanding of mitochondrial DNA (mtDNA), sequence variation in particular mt haplogroups (hgs) and population structure, reliable low-cost high-throughput genotyping assays are needed. Furthermore, methods facilitating sensitive mixture detection and relative quantification of allele proportions are indispensable for the study of heteroplasmy, mitochondrial sequence evolution, and mitochondrial disorders. Here the properties of a homogeneous competitive duplex allele specific PCR (ARMS) assay were scrutinized in the light of these requirements.

**Methodology/Principal Findings:**

A duplex ARMS assay amplifying either the ancestral mtDNA 2706G allele (non-hg H samples) or the derived 7028C allele (hg H samples) in the presence of SYBR Green fluorescent reporter dye was developed and characterized. Product detection, allele calling, and hg inference were based on the amplicon-characteristic melting-point temperatures obtained with on-line post-PCR fluorescent dissociation curve analysis (DCA). The analytical window of the assay covered at least 5 orders of magnitude of template DNA input with a detection limit in the low picogram range of genomic DNA. A set of forensically relevant test specimens was analyzed successfully. The presence of mtDNA mixtures was detected over a broad range of input DNA amounts and mixture ratios, and the estimation of allele proportions in samples with known total mtDNA content was feasible with limitations. A qualified DNA analyst successfully analyzed ∼2,200 DNA extracts within three regular working days, without using robotic lab-equipment. By performing the amplification on-line, the assay also facilitated absolute mtDNA quantification.

**Conclusions:**

Although this assay was developed just for a particular purpose, the approach is general in that it is potentially suitable in a broad variety of assay-layouts for many other applications, including the analysis of mixtures. Homogeneous ARMS-DCA is a valuable tool for large-volume studies targeting small numbers of single nucleotide polymorphisms (SNPs).

## Introduction

The human mitochondrial (mt) genome consists of a small circular chromosome comprising approximately 16,568 base pairs (bp; revised Cambridge reference sequence, rCRS, [1]). Besides the non-coding control region, the mt genome contains the genes for 22 tRNAs and two rRNAs required for intra-organellar translation of the 13 polypeptide encoding mtDNA genes. A diploid human cell (e.g. a nucleated blood cell) usually contains two copies of a particular nuclear marker or gene but hundreds to thousands of mt genomes [2], [3]. Therefore, genotyping of the highly polymorphic control region (or parts thereof) has become a standard tool in forensic genetics, when analyzing biological material containing insufficient amounts of amplifiable genomic DNA (e.g. shed hairs, stains that suffered heavy environmental stress, aged biological material) for the analysis of the highly informative nuclear DNA short tandem repeat markers. Due to its maternal mode of inheritance and the apparent lack of recombination, mtDNA forms stable lineages and haplogroups. Consequently, mtDNA testing does not provide definitive identification of individuals because all members of a maternal lineage are expected to match each other as long as no mutations occur. On the other hand, this makes mtDNA typing a superb tool for forensic human identification when no close relatives are available for comparison. As the weight of a mtDNA match between evidence- and reference-sample depends on the frequency of the found haplotype in the particular (sub)population, a large mtDNA database fulfilling high quality standards is needed for the calculation of the probability of a match by chance, being an alternative explanation for the found haplotype conformity (e.g. [4]).

At large, individuals display - within the detection limits associated to current sequencing technology - only a single mtDNA haplotype (homoplasmy), but individuals can also carry more than just one mtDNA variant, a state known as heteroplasmy (see e.g. [5], [6]). With increasing numbers of analyzed samples as well as improvements in detection chemistries and instrumentation it became clear that heteroplasmy occurs (also in individuals unaffected by mitochondrial disorders) at considerably higher rates than originally assumed [7], [8], [9], [10]. Heteroplasmy not only has the potential to put extra weight on haplotypes by increasing the overall (forensic) information content of a particular haplotype [11], but also plays a paramount role for mtDNA evolution as well as the understanding and diagnosis of mitochondrial diseases. For a number of mitochondrial disorders, the mutant load of the affected cells has to reach a certain cell and tissue type dependent level to show a clinical phenotype [12], and the partition of the (heteroplasmic) mt genomes during cell division is subject to mitotic segregation or a germline bottleneck [13], [14]. Somatic and germline mtDNA mutations were reported to be subject to random drift [15], [16], positive or cleaning selection [17], [18], [19], [20], [21], and rapid evolutionary processes can be observed in cancer [22], [23], [24]. Mitochondrial disorders and mtDNA sequence evolution follow the rules of population genetics at the cellular level [25].

These points clearly emphasize the importance of genotyping approaches capable of sensitive mixture detection and, ideally, relative quantification of allele proportions over a broad range of DNA quality and quantity in both forensic and medical genetics, as well as in studies on molecular mtDNA evolution.

To gain a better understanding of mtDNA variability within a particular haplogroup, heteroplasmy, and population (sub)structure, large scale studies are desirable. However, analysis costs are usually prohibitive to the genotyping of the entire sample set. Therefore, reliable low cost and high-throughput pre-screening methods are needed.

Here we report the results of a validation study on an easy to perform low-cost pre-screening assay developed for the fast exclusion of non-hg H samples in a large West Eurasian population sample to avoid superfluous typing of approximately half of the specimen in a previously published study [26] aiming at a fine resolution within hg H by minisequencing 45 known coding region single nucleotide polymorphisms (SNPs).

## Results and Discussion

### Assay Outline

**Figure 1:**
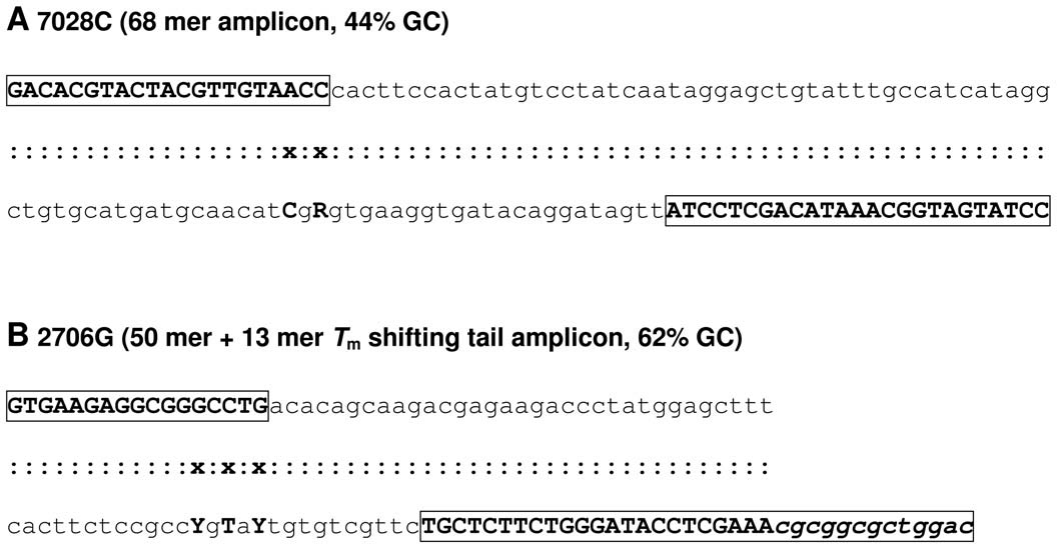
Amplicon and primer sequences. Primer sequences are shown as boxed bold face letters and the melting point temperature shifting 5′ tail attached to primer 2706R is marked by italics formatting. Template strand sequences are shown in lower-case characters in the lower rows of panels (A) and (B), and mismatches are indicated in the H strand sequence by capitalized, non-boxed, bold-face letters.

A homogeneous competitive duplex allele-specific PCR assay (the amplification refractory mutation system, ARMS, [27], [28]) specifically amplifying either the derived (rCRS) 7028C allele (hg H samples) or the ancestral (non-rCRS) 2706G allele (non-hg H samples) in the presence of the non target but dsDNA specific fluorescent reporter-dye SYBR Green I [29], [30] was developed and characterized. ARMS was described shortly after the introduction of PCR and consists solely of allele-specific PCR. It can be used in various assay layouts to detect in principle any known base substitution [31] and (small) insertions/deletions. For each of the two analyzed mtDNA SNPs, the competitive duplex ARMS utilized for both target sequences one non-allele-specific primer and one with a 3′ terminal base specific for the interrogated allelic variant (Figure 1). In principle, the specificity of the reaction depends on the inability of non-proofreading DNA polymerases (e.g. *Taq*) to extend 3′ terminally mismatched oligonucleotides but not on the length of the primers, which makes primer design straightforward by avoiding complex design considerations [32]. However, depending on the nature of the mismatch, *Taq* polymerase tends to extend hybridized primers despite a 3′ terminal mismatch [33], albeit at much lower rates. In order to improve specificity, an additional deliberate mismatch [32] was introduced at the 3′ antepenultimate nucleotide position (ntp) of both allele-specific forward primers (Figure 1). Consequently, these primers displayed a single mismatch with their intended target alleles (C:T at ntp 2704; A:C at ntp 7026), two mismatches with the alternative allelic variant (C:T at ntp 2704 and G:T at ntp 2706; A:C at ntp 7026 and C:A at ntp 7026; Figure 1), and zero mismatches with their PCR products, ensuring both specificity and sensitivity. Allele scoring and haplogroup inferences were based on the determination of the product-characteristic melting point temperature (*T*_m_) values by on-line recording of the decrease in fluorescence-signal during a post-PCR temperature ramp from 60°C to 90°C (dissociation curve analysis, DCA, [29]).

### Reproducibility

To test the intra- as well as the inter-run *T*_m_ variability obtained for hg H and non-hg H samples, two independent experiments were performed on two consecutive days comprising 18 replicates for both the mtDNA hg H and the non-hg H standard.

In 32-cycle ARMS-DCA, using initial total genomic DNA (gDNA) amounts of 10 ng and the 7500 Fast Real-Time PCR System (Applied Biosystems, Foster City, CA), the average melting point temperatures of 7028C (*T*_m_ = 73.6±0.2°C; mean ± standard deviation; n = 36; pooled data from two independent experiments) and 2706G (*T*_m_ = 81.4±0.2°C; n = 36; pooled data from two independent experiments) amplicons differed due to a *T*_m_-shifting, non-homologous, GC-rich tail [34], [35] attached to the 5′ end of the 2706 reverse primer (Figure 1, Figure S1) by 7.8°C from each other, facilitating unambiguous product scoring. The intra-run *T*_m_ variability (18 replicates per run and hg H respectively non-hg H standard sample; Table 1) amounted to less than 1°C for both amplicons, and the average *T*_m_ values obtained in the two independent experiments differed between 0.0°C (7028C amplicons) and 0.1°C (2706G amplicons) from each other (Table 1).

**Table 1:** ARMS-DCA reproducibility.

Experiment	1	2	1	2
**Amplicon**	7028C	7028C	2706G	2706G
**Average ** ***T*** **_m_**	73.6°C	73.6°C	81.3°C	81.4°C
**Standard Dev.**	0.2	0.3	0.3	0.2
**Replicates**	18	18	18	18
**Min. ** ***T*** **_m_**	73.1°C	73.3°C	81.1°C	80.9°C
**Max. ** ***T*** **_m_**	73.8°C	74.0°C	81.8°C	81.6°C
***T*** **_m_ Range**	0.7°C	0.7°C	0.7°C	0.7°C

The variability of 7028C and 2706G amplicon melting point (*T*_m_) values was evaluated in two independent experiments performed on two consecutive days (n = 18 real replicates per hg H respectively non-hg H standard gDNA sample and experiment, 32 cycle ARMS-DCA, 7500 Fast Real Time PCR system).

### Sensitivity and Dynamic Range

**Figure 2:**
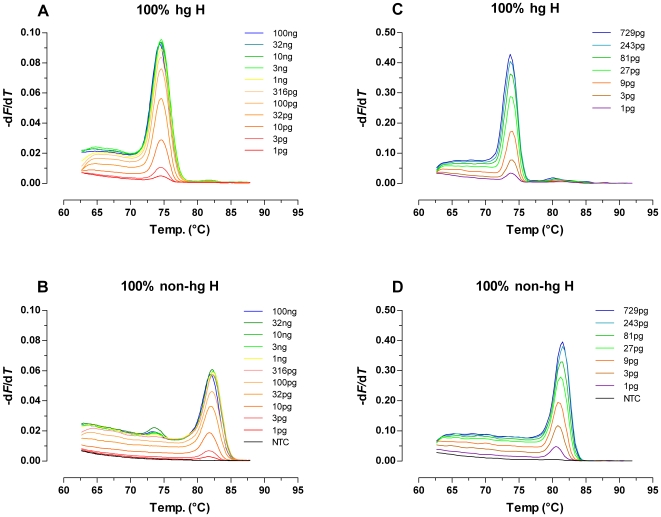
Analytical window of 32-cycle ARMS-DCA for hg H and non-hg H templates. (A, B) Melt-peak data shown originated from an experiment on an ABI PRISM 7700 Sequence Detector. (C, D) Data shown originated from a sensitivity check performed on an Applied Biosystems 7500 Fast Real Time PCR System. Total genomic DNA input amounts are indicated in the panels. For all experiments, 1 pg gDNA equalled approximately 100 mtGE.

Initial experiments showed that limiting the number of ARMS PCR cycles to 32 yielded the best trade-off between sensitivity and specificity. Being an end-point method, the optimal setting for this assay system might, however, vary with different (real-time PCR) instruments and/or SYBR Green I detection chemistries from other suppliers (or even different lots of the same detection chemistry), and the primer batches used. Experiments performed on a 7500 Fast Real-Time PCR System were characterized by higher melt-peaks resulting from higher endpoint fluorescence signals (after normalization to the passive internal reference dye ROX) when compared to data from an ABI PRISM 7700 Sequence Detector (both Applied Biosystems; Figure 2). Nevertheless, with both real-time PCR instruments the analytical window of 32-cycle ARMS-DCA covered for both hg H and non-hg H templates almost the entire range of initial gDNA amounts tested (1 pg–100 ng; for both gDNA standards used in the study 1 pg equalled approximately 100 mitochondrial genome equivalents, mtGE; Figure 2), enabling the analysis of a broad spectrum of samples with a single assay regardless of DNA concentration.

### Mixture Detection

For the mtDNA haplogroup H4a an A to G back-mutation at ntp 2706 was reported by [36]. Thus, with the ARMS-DCA approach the analysis of hg H4a mt genomes would result in melt-peaks for both target sequences. The same is true e.g. for mixed genetic material found at a crime scene (“stain”) comprising hg H and non-hg H mtDNA contributions. Contrary to the backmutation in hg H4a, resulting in a 1∶1 pseudo-mixture, heteroplasmic samples as well as multi-contributor stains have the ability to display allele proportions on a continuous scale.

**Figure 3:**
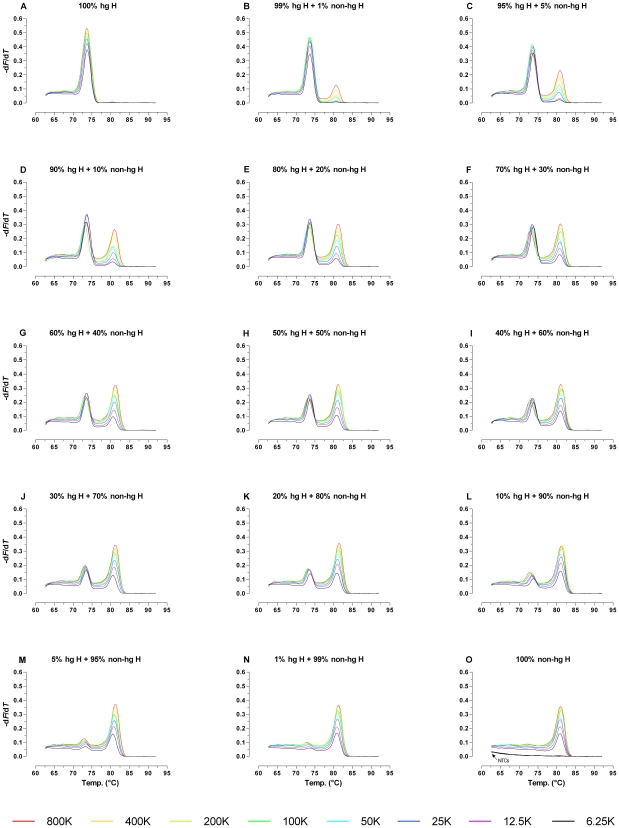
Sensitivity of 7028C/2706G mixture detection depending on the initial amount of template molecules. Data shown originate from two independent 32-cycle ARMS-DCA experiments (800,000–100,000 mtGE and 50,000–6,250 mtGE, n = 1 per mixture ratio/mtDNA input combination) on a 7500 Fast Real Time PCR System. Total template molecule input amounts are given in mtGE, K: ×1000; 1 mtGE equals approximately 10 fg gDNA standard; NTC: no template control.

To evaluate the assay's performance regarding mixture detection and relative allele-quantification, duplex competitive ARMS-DCA was tested on a series of defined mixtures of two sequence verified single donor gDNA samples belonging to either hg H* or hg J2b1a at total mtDNA input amounts ranging from 6,250 to 800,000 mtGE (approx. 62.5 pg – 8 ng gDNA standard). Regardless of the initial amount of DNA 5% hg H contributions generated clearly visible melt-peaks (Figure 3). However, when using 32 cycles of amplification and very high DNA input amounts (e.g. 100 ng) sometimes small non-specific 7028C melt-peaks were observed in addition to the specific 2706G signal for non-hg H samples (Figure 2). Therefore, a practical threshold of 10% appears to be appropriate for the detection of minor hg H components when analyzing unknown samples with a very high (expected) DNA content (Figure 3). For 2706G alleles, the detection limits for minor contributions below 10% were depending on the total DNA input (Figure 3). Well-defined melt-peaks were observed at the 1% and 5% non-hg H level for reactions initially containing at least a total of 100,000 respectively 25,000 mtGE (approximately 1 ng respectively 250 pg gDNA; Figure 3).

### Relative Quantification of Mixture Ratios

**Figure 4:**
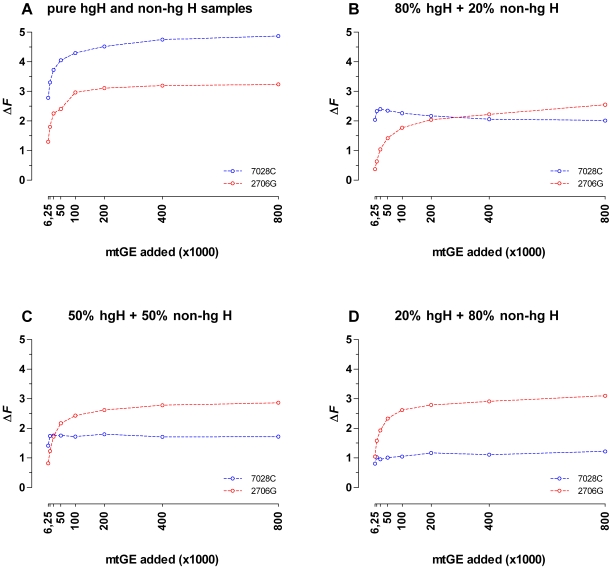
mtDNA-input dependence of 7028C and 2706G melt ramp heights in pure and admixed hg H and non-hg H samples. The heights of the 7028C and 2706G melt-ramps (Δ*F*) were determined from non-normalized melting curves (32-cycle ARMS-DCA; 7500 Fast Real Time PCR System) obtained in two independent 32-cycle ARMS-DCA experiments (800,000–100,000 mtGE and 50,000–6,250 mtGE, singleton data points).

Being easily obtainable, 2706G and 7028C melt-peak information was utilized in a pilot attempt to quantify mixture ratios [37]. However, this was complicated because at a given mixture ratio (see e.g. Figure 3E: 80% hg H +20% non-hg H) the 2706G peaks revealed a distinct increase in height with increasing mtDNA input amounts, whereas the 7028C melt-peaks remained nearly unaffected. This caused a pronounced dependency of the 7028C/2706G melt-peak height ratios on the mtDNA input amount at a given hg H/non-hg H mixture ratio. The width of the temperature-range over which amplicon-melting occurred revealed no obvious dependency on the initial template concentration (Figure 3). Therefore, the mtDNA input amount dependent differences in the PCR product related maximum rates of fluorescence-change with temperature (i.e. the heights of the negative derivative melt-peaks) had to result mostly from an effect on the dissociation curve ramp heights (Δ*F* 2706G or Δ*F* 7028C; melt-ramp heights; Figure S1A), which are a measure for the net-amount of liberated SYBR Green I dye per amplicon species during its transition from the double stranded to the random coiled state. For pure hg H and non-hg H samples, the Δ*F* 7028C respectively Δ*F* 2706G values showed a distinct mtDNA input amount dependent decline below ∼200,000 mtGE/reaction (Figure 4). The 2706G melt-ramp heights obtained for the examined mixtures followed the same pattern (Figure 4). Contrary, Δ*F* 7028C values obtained for serial dilutions of mixed hg H and non-hg H samples revealed a pronounced change in their response to lowered amounts of initial template molecules as compared to the results for pure hg H samples. Except for the lowest mtDNA input amount (6,250 mtGE/20µl reaction) Δ*F* 7028C values became in all mixtures nearly independent from the number of the added template molecules (Figure 4). These findings clearly mirrored the observed effects of mtDNA input amounts on the 7027C and 2706G melt-peak heights. For the 80% hg H +20% non-hg H mixture, the 800,000 mtGE/reaction 7028C melt-ramp was more than twofold reduced in height as compared to the pure hg H sample, and for the other mixtures, comprising lower hg H proportions, even lower values were obtained for Δ*F* 7028C (Figure 4). This effect was almost unobservable for the Δ*F* 2706G values in all three analyzed mixtures.

**Figure 5:**
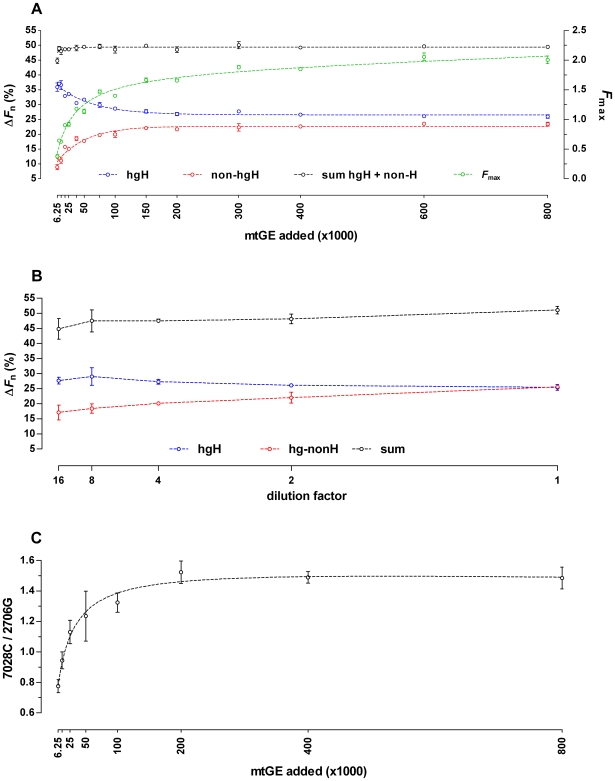
mtDNA-input dependence of Δ*F*_n_ and *F*_max_, dye translocation, and preferential amplification. (A) The Δ*F*_n_ and *F*_max_ data (n = 3, mean ± standard deviation) obtained for an 80% hg H +20% non-hg H mixture originated from two independent 32-cycle ARMS-DCA experiments (800,000 and 600,000 mtGE down to 6,250 and 9,375 mtGE per reaction, respectively, in twofold dilution steps) on a 7500 Fast Real Time PCR System. (B) No template control reactions were used for serially diluting the PCR product (initial template amount in reaction: 800,000 mtGE; 80% hg H +20% non-hg H target-mixture; 32-cycle ARMS-DCA; 7500 Fast Real Time PCR System; data from two independent experiments, n = 5, mean ± standard deviation) to test for amplicon concentration dependent effects on Δ*F*_n_ 7028C and 2706G. (C) 7028C and 2706G band intensities were determined after polyacrylamide gel-electrophoretic separation of PCR products followed by silver staining (80% hg H +20% non-hg H target-mixture; 32-cycle ARMS-DCA, single experiment, n = 3, mean ± standard deviation).

**Figure 6:**
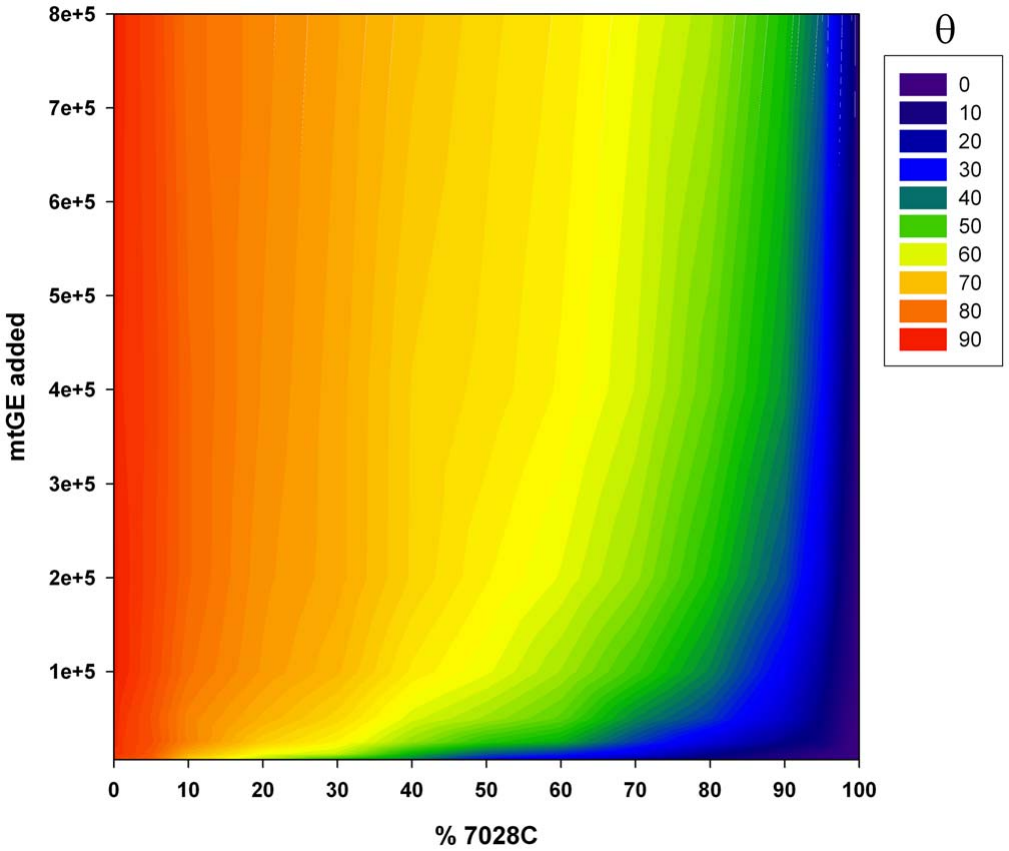
Influence of the initial copy number and mixture-ratio on the 7028C/2706G melt-ramp height ratio. The ratios of normalized 7028C and 2706G melt-ramp heights (2 independent experiments: 800,000–100,000 mtGE and 50,000–6,250 mtGE, singleton data points, 32-cycle ARMS-DCA; 7500 Fast Real Time PCR System) were visualized as color-coded angles (θ) in degrees.

**Figure 7:**
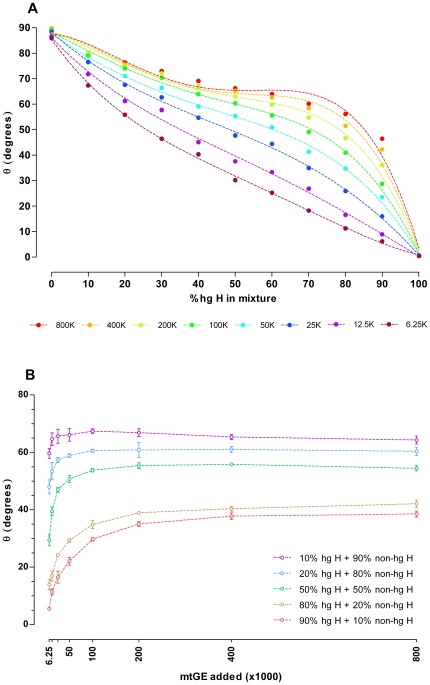
Mixture ratio resolution and reproducibility of θ values. (A) The ratios of normalized 7028C and 2706G melt-ramp heights (2 independent experiments: 800,000–100,000 mtGE and 50,000–6,250 mtGE, singleton data points, 32-cycle ARMS-DCA; 7500 Fast Real Time PCR System) obtained for pure and admixed hg H and non-hg H templates were transformed into an angle θ and expressed in degrees. Total template molecule input amounts are given in mtGE; K: ×1000. (B) θ values for the selected hg H non-hg H admixtures were obtained with 32-cycle ARMS-DCA (7500 Fast Real Time PCR System; 80% hg H+20% non-hg H: single experiments, n = 3, mean ± standard deviation shown).

End-point PCR product yields vary markedly for samples comprising largely different amounts of initial template molecules, especially when the plateau phase of the amplification is not reached within the number of applied cycles. Thus, in order to make melt-ramp height based analysis results easier to compare, the dissociation curves were normalized to their highest (100%) and lowest (0%) fluorescence readings. As expected, also the analysis of normalized dissociation curves revealed a pronounced dependency of the Δ*F*_n_ values – representing the relative contributions of the 2706G and 7028C amplicons to the highest observed overall reporter signal (*F*_max_) during DCA (Figure 5A; Figure S1) – on the used mtDNA input concentration. When analyzing e.g. the 80% hg H +20% non-hg H mixture with 32-cycle ARMS-DCA, the lowest initial DNA concentration tested (6,250 mtGE/20µl assay) yielded the lowest *F*_max_ value (i.e. the least PCR product) and consequently the highest concentration of free SYBR Green I (Figure 5A). Under these conditions (and ignoring the direct temperature effect on the reporter dye) 36% of *F*_max_ originated from the 7028C amplicons and 9% could be attributed to the 2706G products, being the highest respectively lowest values found at this mixture ratio for all tested input DNA concentrations (Figure 5A). With increasing template DNA concentration, however, both the *F*_max_ and the Δ*F*_n_ 2706G values increased hyperbolically (maximum Δ*F*_n_ 2706G: 26% at 800,000 mtGE/20µl reaction), whereas the Δ*F*_n_ 7028C values decreased to the same degree (min. Δ*F*_n_ 7028C: 23% at 800,000 mtGE/20µl reaction), keeping the sum of Δ*F*_n_ 7028C plus Δ*F*_n_ 7028C nearly constant at 48–50% for DNA input amounts of 12,500 mtGE or higher (Figure 5A). Figure S2 summarizes the results obtained in a series of two independent experiments for various mixture ratios with different DNA input amounts. With competitive duplex ARMS-DCA of hg H/non-hg H mixtures DNA input dependent preferential binding of SYBR Green I under PCR conditions to the longer PCR products or those with the higher GC-content [38] was unlikely to explain these observations, as for pure target DNA preparations Δ*F*_n_ values of 30%–41% and 56%–64% were found, respectively, for 2706G (63 bp, 62%GC) and 7028C (68 bp, 44%GC; Figure 1) amplicons over the entire input DNA concentration range tested (Figure S2A). Another explanation for the effect of the DNA input amount on the melt-peak height ratio could be dye translocation [38], [39], [40], causing a decreased net-release of reporter fluorochrome due to an amplicon-concentration dependent redistribution of dye molecules liberated by the melting of the lower *T*_m_ amplicons (7028C) to the higher *T*_m_ products (2706G). Dye translocation between the two PCR products is possible because SYBR Green I cannot be used at saturating concentrations that cause PCR inhibition [30], [41], [42]. The higher the initial DNA concentration – causing increasing PCR product yields (fluorescence signals, *F*_max_) as long as the number of applied amplification cycles is a limiting factor (Figure 5A) – the more dye molecules are liberated by the post-PCR strand dissociation of the 7028C amplicons, and consequently, the higher the increase in free SYBR Green I will be, causing increased dye binding to the still double stranded 2706G products when the amount of SYBR Green I present is limiting. Therefore, the dye translocation effect on the net-liberation of reporter fluorochrome from the lower *T*_m_ product should be most pronounced at the highest DNA input amounts and (almost) absent at the lowest initial template molecule concentrations used. This proposition was compatible with the experimental data. To test this hypothesis, a dilution series of the 80% hg H +20% non-hg H mixture ARMS products was prepared using no template control reactions as diluent, and analyzed by DCA. The *F*_max_ value of the 6,250 mtGE reaction fell between those measured for the 8- and 16-fold dilutions. As shown in Figure 5B, Δ*F*_n_ 2706G decreased with decreasing product concentrations, whereas the Δ*F*_n_ 7028C values slightly increased until a dilution factor of eight. While providing strong evidence for amplicon-concentration dependent 7028C to 2706G dye translocation at a fixed proportion of the two ARMS products, these results failed to fully explain the observed effect of the initial DNA concentration on the melt-peak height ratios and the Δ*F*_n_ values (Figure 5A, Figure 5B). When visualizing the 80% hg H +20% non-hg H ARMS products by polyacrylamide gel electrophoresis and silver staining, preferential amplification of 2706G over 7028C targets with increasing total target molecule concentrations was observed (Figure 5C). Consequently, the combined effects of preferential amplification and dye translocation are the most likely explanation for the correlation of the melt-peak height ratios with the template DNA input amounts. As summarized in Figure 6, the interrelation between mixture ratio, DNA input amount, and obtained Δ*F*_n_ 7028C/2706G ratios (transformed into an angle θ and expressed in degrees; θ = 0°: pure hg H sample; θ = 90°: pure non-hg H sample), puts a constraint on DCA based mixture ratio assessment for samples with unknown DNA content, since equal θ values were obtained for different mixture ratios depending on the initial template molecule concentration. At the lower end of mtDNA input amounts tested, θ showed the highest dependency on initial copy numbers and the best resolution between different mixture ratios was obtained (Figure 6, Figure 7A). Contrary, θ became nearly independent from the initial amount of template molecules when ARMS reached the plateau phase of amplification within 32 PCR cycles (mtDNA input: ≥200,000–400,000 mtGE/reaction; see *F*_max_ in Figure 5A; Figure 6) but showed only poor resolution between different mixture ratios (Figure 7A). As the resolution is best but the error due to imprecise quantification [43] and/or sample normalization will be largest at the lower end of usable mtDNA concentrations, for reliable assessment of hg H/non-hg H contributions in unknown samples from θ, multiple sample replicates (Figure 7B), accurate quantification of the total mtDNA content, careful adjustment of the total template mtDNA input per reaction, and a calibration curve comprising a series of different mixture ratios at the chosen total initial mtDNA quantity are required.

On the one hand these results demonstrate in principle the assay's suitability for relative quantification of mixture ratios, but on the other hand the associated methodological requirements unfortunately impede (manual) high throughput screening.

### Forensically Relevant Samples and Model Inhibitors

In forensic casework analysts frequently have to deal with both samples comprising high (e.g. reference material or well preserved large blood stains) and very low amounts of DNA. Typical examples for the latter group are e.g. micro-stains, swabs from touched items, or samples that suffered from DNA degradation due to unfavourable environmental conditions and/or storage over prolonged time spans. The high sensitivity of the duplex ARMS-DCA approach and the short minimum template molecule lengths required for amplification (2706G: 50 nt; 7028C: 68 nt; Figure 1) along with the wide analytical window and the ability to detect mixtures, rendered the ARMS-DCA assay a good candidate for the analysis of samples frequently encountered in forensic genetics. As depicted in Figure S3 this could be verified in principle by the successful typing of gDNA extracted from a test-panel of nonprobative forensically relevant samples comprising simulated single- and multi-contributor crime scene stains, such as hairs, cigarette butts, chewing gum, swabs from touched items, and old teeth, as well as reference samples (buccal swabs).

**Figure 8:**
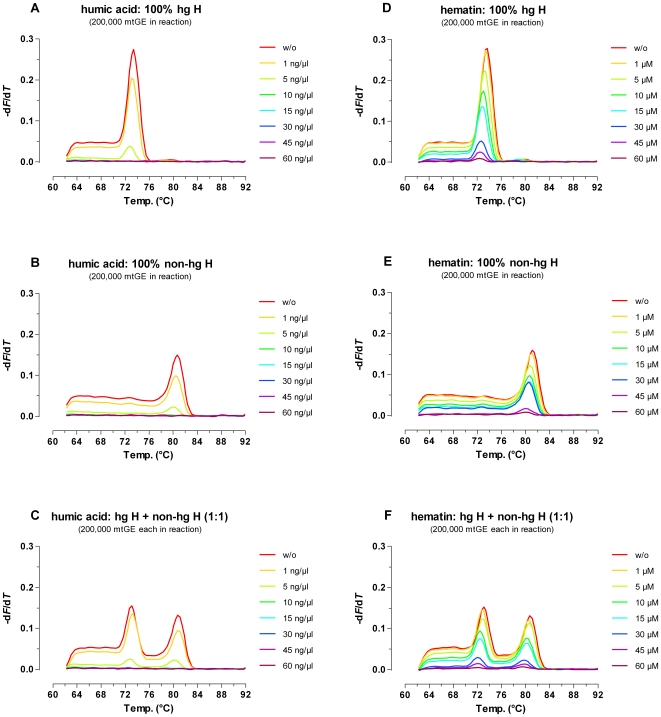
Effects of model inhibitors. Humic acid (A–C) and hematin (D–F) were used as model substances to assess the effects of PCR inhibitors on ARMS-DCA typing of pure and admixed hg H and non-hg-H samples (32-cycle ARMS-DCA, 7500 Fast Real Time PCR System).

Moreover, poor-quality of the template DNA can impact PCR success and/or amplicon melt-characteristics. Real world samples (e.g. evidence material collected at a crime scene) frequently contain PCR inhibiting substances that might be unintentionally co-purified with the DNA during extraction. In order to evaluate the effects of PCR inhibitors on ARMS-DCA typing of pure and admixed hg H/non-hg H samples (200,000 mtGE/reaction), hematin [44], [45] and humic acid [46] were used as model substances. When exceeding an assay concentration of 1 µM, hematin caused a graded decrease of the melt-peak heights for both interrogated SNP alleles. Unambiguous hg H/non-hg H assignments of pure samples as well as detection of a 1∶1 hg H/non-hg H mixture (200,000 mtGE each/reaction) were possible up to 30 µM hematin, whereas at higher inhibitor concentrations no (60 µM) or only very weak melt-peaks (45 µM hematin) were observable with 32 cycle ARMS-DCA (Figure 8). Humic acid, the second model inhibitor used, mirrored the effects observed for hematin. Assay concentrations of 1 ng/µl and 5 ng/µl caused clear respectively very strong drops of the melt-peak heights for both, pure hg H and non-hg H standard samples as well as the 1∶1 mixture thereof (Figure 8). At concentrations of 10 ng/µl or higher, humic acid caused complete failure of amplification. Thus, for pure single contributor samples as well as balanced hg H + non-hg H mixtures increasing concentrations of both model inhibitors apparently reduced the assay sensitivity for both targeted SNP alleles to the same degree. For unbalanced mixtures, however, preferential loss of the minority component will occur, depending on the mixture ratio, the total amount of initial template molecules in the reaction, and the concentration of the PCR inhibitor.

The cause for the higher susceptibility of the ARMS-DCA assay to hematin and humic acid as compared to e.g. autosomal STR typing or quantitative real-time PCR using TaqMan Chemistry [3], [46] lies probably in the deliberately introduced mismatches with the template mtDNA targets and the rather low number of applied PCR cycles as compared to quantitative real-time PCR approaches. These findings highlight the paramount importance of avoiding carryover of substances negatively interfering with the intended downstream application(s) by using appropriate DNA extraction methods. Recent advances in PCR chemistry [47], [48], [49] mediated significant improvements in the robustness of autosomal STR typing assays by overcoming some of the adverse effects of PCR inhibitors. Obviously, an according update of existing real-time PCR chemistries would be beneficial, not only for ARMS-DCA but also e.g. quantitative real-time PCR assays containing internal amplification control systems as testers for the potential presence of PCR inhibitors in DNA extracts and predictors for their effects on downstream (next generation) genotyping assays.

### Absolute mtDNA Quantification Using Real-Time ARMS

Accurate knowledge of the target molecule concentration in unknown samples not only increases the chances for successful genotyping but also provides valuable information for the assessment of the reliability of the results obtained for low copy number samples. On-line monitoring of the accumulation of amplicons formed in the competitive duplex ARMS in a real-time PCR machine (rtARMS) enabled mtDNA quantification over a wide linear dynamic range (100–1,000,000 mtGE/20µl reaction) and revealed apparent single-cycle PCR efficiencies during the exponential reaction-phase of 98.3% (95% confidence interval: 96.5–100.2%; 7028C) and 106.4% (101.5–111.8%; 2706G). The slopes of the calibration curves for 2706G: (y = −3.177 log_10_(x) +30.15, r^2^ = 0.9905) and 7028C (y = −3.363 log_10_(x) +30.54, r^2^ = 0.9985) differed significantly (*p* = 0.002, ANCOVA). Thus, the calculation of a common calibration curve for both qrtARMS targets was not feasible. This has to be kept in mind when interpreting total mtDNA quantification results for samples displaying both product specific melt-peaks.

### Population Study and Potentially False Haplogroup Assignments

A single analyst successfully analyzed a West Eurasian population sample comprising 2,214 DNA extracts in three regular working days, using a multiple dispensing device with interchangeable tips, an 8-channel pipette, and manual allele-scoring. All but 12 (0.5%) DNA extracts in this population sample could be typed unambiguously, attributing 863 samples to hg H (39.0%) and 1,339 samples to the non-hg H pool. Except for 4 low DNA quality samples that yielded insufficient amounts of PCR products for downstream SNaPshot mtDNA SNP genotyping, all hg H inferences were verified by minisequencing of mtDNA coding region SNPs and Sanger-type mtDNA control region sequencing [26], [50]. The dissociation curves of all 12 samples with ambiguous allele scores displayed very low 7028C and no 2706G melt-peaks (data not shown) and were typed non-hg H by minisequencing [26]. Sanger type sequencing revealed for all of them the 2706G/7028T allelic states and an additional G to A transition in the 2706G_F primer binding site at ntp 2702. The 2702A allele was reported by [51] for the mtDNA haplogroup N1a. With 32-cycle ARMS-DCA and high initial amounts (e.g. 100 ng gDNA/reaction) of these non-hgH 7028T/2706G/2702A templates, preferential non-specific amplification with the 7028C primer pair was observed (Figure S1), resulting in potential miss-assignment of these samples to hg H. Therefore, the alternative allele-specific forward primer 2706G_F* (GTGAAGAGGCGGACCTG) was designed. Equimolar inclusion of this primer (150 nM) in the ARMS-DCA assay yielded correct allele calls for the 12 samples under scrutiny and completely suppressed detectable non-specific 7028C product formation, without observable negative effects on the typing of pure and deliberately admixed 7028C/2706A and 7028T/2706G samples (26- and 32-cycle ARMS). Consequently, the actual number of non-hg H samples in the Austrian population sample amounted to 1,351 DNA extracts (61.0%) [26].

Samples typed as non-hg H in the population study were not further analyzed in [26]. Therefore, false negative haplogroup assignments cannot be fully excluded. A search of an in-house database containing 3,571 published full mt genome sequences for additional sequence variation in the 7028C forward and reverse primer-binding sites revealed 2 SNPs that might have prevented hybridization with hg H targets and making 7028C amplification impossible. 3 G7013A transitions in 649 hg H samples (0.46%) were found by [52] and 1 individual carrying T7058C was reported for a set of 545 hg H samples (0.18%) [53]. However, the competitive duplex ARMS-DCA assay appears robust against erroneous non-hg H sample scores due to sequence variation in the 7028C primer binding sites. In a set of 90 population study samples, none of the 26 known to belong to hg H [26] yielded detectable melt-peaks with singleplex 2706G ARMS-DCA under the experimental conditions used for the population study (data not shown). Thus, erroneous allocation of samples to the non-hg H sample pool due to non-specific 2706G product formation on 2706A/7028C targets under non-competitive PCR conditions (lack of 7028C amplification) appears unlikely.

### Conclusions

Here we report on properties of an easy to perform low-cost homogeneous competitive duplex ARMS-DCA assay for the scoring of mtDNA hg-H/non-hg H samples by interrogating the mtDNA 7028C/2706G SNP-alleles. Features like the lack of post-PCR manipulations, high specificity, sensitivity, and reproducibility made this high-throughput assay a valuable tool.

ARMS-DCA enabled the successful analysis of the entire Austrian population sample comprising 2,214 DNA extracts within 3 regular working days without the use of robotic lab-equipment. This impressively illustrated the sample-throughput capabilities of ARMS-DCA. Furthermore, if needed, the number of simultaneous analyses could be quadruplicated by simply switching from the 96-well to the 384-well thermal cycler block format, and adoption of robotic lab-equipment together with automated routines for allele scoring would make very high sample-throughput possible.

The wide analytical window of ARMS-DCA enabled reliable SNP typing and absolute mtDNA quantification over a broad range of input DNA amounts with a single experimental setup. The short required amplicon lengths accounted for high PCR efficiencies and fostered the analysis of forensically relevant sample-types including swabs from contact stains and degraded DNA extracted from old teeth.

The detection of mtDNA hg H and non-hg H mixtures was possible over a wide range of mixture ratios and initial DNA concentrations. The ARMS-DCA assay was shown to facilitate in principle the determination of allele proportions on the basis of the observed Δ*F*_n_ 7028C/2706G ratios. However, DNA input amount dependent preferential amplification together with amplicon-concentration dependent dye translocation considerably constrict high throughput endpoint-fluorescence based relative quantification of allele proportions.

Another confinement that applies to ARMS-DCA lies in its limited multiplexing capability. While the feasibility of e.g. quadruplex SNP typing was successfully demonstrated for both ARMS [54] as well as high resolution amplicon melting [35], the simultaneous analysis of large SNP panels will not be possible with this approach.

The adverse effects exerted by an additional G>A sequence variation in the binding site of the allele-specific 2706G forward primer at ntp 2702 could be easily overcome by the inclusion of an alternative primer. However, this solution would clearly fail when multiple primer binding sites are affected, a scenario that is likely for highly variable sequence regions such as the mitochondrial control region. Here, alternative solutions such as switching the template strand to avoid base substitutions close to the 3′ end of the allele-specific primer and/or lengthening it to overcome the hybridization destabilizing effects of additional mismatches with the more central part of its binding sequence might also be insufficient. As with other currently used mtDNA typing methods such as sequencing analysis of multiplexed short PCR products, hybridization probe based assays, or minisequencing, successful assay designs might be challenging or even impossible for particular sites in such “unstable” sequence environments.

Yet, ARMS-DCA assay development is easy (for “stable” sequence neighbourhoods) and does not require large investments of time, money and labour. This renders ARMS-DCA – despite its limitations – a good choice for developing assays facilitating e.g. the pre-screening of large population samples in order to identify the relevant DNA extracts or the fast and cost-efficient mtDNA based exclusion of innocent individuals in DNA dragnets.

Although we described and characterized ARMS-DCA here just for a particular purpose, the approach is general in that it is suitable in a variety of assay-layouts for many other applications. We successfully applied ARMS-DCA to the fast discrimination between mtDNA hg K and non-hg K samples (total n = 3,680) by simultaneously targeting the 9055A and G alleles, respectively, and the subsequent multiplexed subtyping for hg K1 (1189C) vs. K2 (9716C) vs. potential K* (lack of K1, K2 products but amplification of an internal mtDNA control target) individuals (manuscript in preparation).

Another application for ARMS-DCA results from its ability to selectively amplify rare mutant alleles in a background of wild-type sequences, a feature being of paramount importance in cancer screening. In this laboratory, ARMS-DCA was used in a previous study to identify samples carrying a nonsense mutation in the repetitive Kringle IV-2 domain of the human gene for apolipoprotein (a) [55].

Both ARMS as well as dissociation curve analysis are “classics” for the analysis of sequence variation. However, considerable progress is taking place regarding homogeneous genotyping approaches relying on amplicon melting to distinguish genetic variants. Novel dsDNA binding dyes that can be used at saturating concentrations (e.g. [29], [30], [42]) and advanced instruments together with dedicated software solutions (e.g. [56]) allow for high resolution amplicon melting with largely reduced inter-sample/well-to-well melt-variability, that can be further reduced by calibration of the melt-curve data to spiked oligonucleotide melt-standards [57]. Short amplicon DNA melting enables the creation of customized positive control samples for assays targeting rare alleles by simply making use of a commercial oligonucleotide or gene synthesis service.

Considerable progress is also made in the development of novel types of oligonucleotides allowing successful primer design in e.g. AT-rich sequence environments (e.g. [58]) and the incorporation of nucleotide analogues into allele-specific primers was shown to further increase their allele-discriminatory potential (e.g. [59]). Together with the gain in convenience, data security, speed, and sensitivity already achieved by implementing standard real-time PCR technology, these recent developments clearly demonstrate that ARMS-DCA is still a state of the art technique.

## Materials and Methods

### DNA Samples and Extraction

Two single donor high molecular weight mtDNA hg H (hg H*) and non-hg H (hg J2b1a) gDNA standards were extracted from peripheral blood from healthy West Eurasian donors using the QIAamp DNA Blood Maxi kit (Qiagen, Hilden, Germany), mtDNA control region sequence verified, and quantified according to [3]. The anonymized DNA samples in the Austrian population study (n = 2,214) were the same as used in [26]. DNA was extracted from the simulated single- and multi-contributor crime scene using a BioRobot M48 Workstation and the MagAttract DNA Mini M48 kit (both Qiagen). Sample preparation and DNA extraction from old teeth followed the procedures described in [60], [61]. Nucleotide position numbers given correspond to the rCRS [1]. Mitochondrial haplogroup allocations were based on build 4 of the phylogenetic tree published by [62].

### Hematin and Humic Acid Inhibitor Models

Porcine hematin (Sigma-Aldrich, St. Louis, MO) was dissolved in a small volume of 1 N NaOH and brought to a concentration of 250 µM by the addition of 10 mM Tris/HCl (pH 8.0). A 600 ng/µl stock solution of humic acid (Carl Roth, Karlsruhe, Germany) was prepared in 10 mM Tris/HCl (pH 9.0). Water was used as diluent for the preparation of the 4x working stocks of both model inhibitors. The final assay concentrations were 0 µM, 1 µM, 5 µM, 10 µM, 15 µM, 30 µM, 45 µM, and 60 µM for hematin, and 0 ng/µl, 1 ng/µl, 5 ng/µl, 10 ng/µl, 15 ng/µl, 30 ng/µl, 45 ng/µl, and 60 ng/µl for humic acid.

### Primer Design

Primer design as well as checks for hairpin formation, self- and cross-hybridization, BLAST searches, and computations of the effects of single base mismatches on the oligonucleotide *T*_m_ were performed with the on-line PrimerQuest and OligoAnalyzer programs (both Integrated DNA Technologies, Coralville, IA, USA; http://www.idtdna.com/SciTools/SciTools.aspx). All primers were HPLC purified by the supplier.

### Competitive Duplex ARMS

For assay development and validation, the individual reaction mixtures were set up in 96-well polypropylene plates in a total volume of 20 µl comprising 1x SYBR Green PCR master mix or 1x Power SYBR Green master mix (both Applied Biosystems), 5 µg non-acetylated BSA, 300 nM each 7028C primer (7028C_F: ntps 7008–7028; 7028_R: ntps 7075–7051; Figure 1), 150 nM each 2706G primer (2706G_F: ntps 2690–2706; 2706_R: ntps 2739–2717; Figure 1), and 1 pg to 100 ng of total gDNA sample. Amplifications were carried out on conventional thermal cyclers or real-time PCR instruments (ABI PRISM 7700 Sequence Detector or 7500 Fast Real-Time PCR System, both Applied Biosystems) by an initial denaturation step at 95°C for 10 min, followed by 32 cycles of denaturation at 95°C for 15 s and annealing/extension at 60°C for 1 min. Standard ramp-speed settings were used with all thermal cyclers. In all ARMS-DCA runs, no template control reactions were included to check for potential contamination of the PCR reagents and consumables. Experiments using a thermal cycler protocol and PCR chemistry formulated for fast thermocycling yielded unsatisfactory results and were therefore discontinued.

For the typing of a West Eurasian population sample from Austria comprising 2,214 DNA samples, off-line ARMS was performed on nominally 40 ng gDNA in conventional thermal cyclers. The same PCR conditions as described for assay development applied, but the reaction volume was reduced to 10 µl and the number of PCR cycles was set to 26 to reduce assay costs and cycler time needed. Storage of set-up reactions at ambient temperature in a drawer over night showed no deleterious effects on the assay (data not shown).

### Post-PCR Dissociation Curve Analysis

Following amplification, the SYBR Green I fluorescence *F* (usually normalized to the passive reference dye ROX) was recorded on the ABI PRISM 7700 Sequence Detector during a 10 min post-PCR temperature ramp from 60°C to 90°C following denaturation at 95°C for 15 s and a fast ramp to 60°C, and with the 7500 Fast Real-Time PCR System the pre-set standard dissociation stage (95°C for 15s, 60°C for 1 min, slow ramp to 95°C) was used. Manual scoring of the PCR products and haplogroup inferences were based on the characteristic *T*_m_ values of the two amplicons. *T*_m_ values were derived from the apexes of the melt-peaks obtained from dissociation diagrams by plotting the negative rate of fluorescence-change with temperature (-d*F*/d*T*) vs. the temperature (Figure S1). For analysis of the raw fluorescence data and their automated conversion into dissociation curve diagrams and melt-peaks [29], either the Dissociation Curves 1.0 software (ABI 7700) or the 7500 Fast System SDS 1.4 software was used (both Applied Biosystems). In initial experiments, immediate post-ARMS DCA and re-DCA after storage of reactions in the dark at room temperature over night or at 4°C for up to 2 weeks yielded consistent genotyping results (data not shown).

### Quantitative Real-Time ARMS

For quantitative real-time ARMS (qrtARMS) experiments, the real-time PCR machines were used for amplification. Product verification and scoring were performed by DCA. Standard curves were constructed using the mtDNA hg H and non-hg H gDNA standards by plotting the threshold cycle values vs. the common logarithm of the initial number of template molecules. Mitochondrial DNA quantities were expressed as mitochondrial genome equivalents (1 mtGE equals 2 single stranded template molecules with a length equal to or exceeding that of the respective ARMS product). The apparent single cycle PCR efficiencies during the exponential phase of the amplification were derived from the slopes of the calibration curves as E = 10^−1/slope^−1 and expressed as a percentage. Depending on the type of real-time PCR instrument used, either the ABI PRISM Sequence Detection Systems 1.9.1 software or the 7500 Fast System SDS 1.4 software (both Applied Biosystems) were used for data acquisition and calculation of the threshold cycle values.

### Mixture Study

A panel of defined mixtures (100%, 99%, 95%, 90% down to 50% major hg H or non-hg H component in steps of 10%) of the two single donor mtDNA hg H* and hg J2b1a gDNA standards was prepared. Two-fold serial dilutions of these pure and admixed samples – resulting in total input amounts of 800,000–6,250 mtGE per 20 µl assay – were amplified and analyzed in a 7500 Fast Real-Time PCR System by ARMS-DCA. Raw dissociation curves (*F* vs. *T*) were normalized by setting their highest and lowest fluorescence signal values to 100% and 0%, respectively (Figure S1). Heights of the steep drops in signal strength (“melt ramps”; Δ*F*_n_ 2706G or 7028C; Figure S1) were determined manually after plotting the normalized dissociation curves (*F*_n_ vs. *T*) on millimeter paper. 7028C/2706G melt ramp height ratios derived from normalized dissociation curves (Figure S1) were transformed into an angle θ by using the “ARCTAN2” function in Microsoft Excel 2002 and expressed in degrees.

Polyacrylamide gel electrophoresis and rapid silver staining of ARMS products were performed according to [63]. Bands were photographed on a light table with a standard digital single-lens reflex camera using the RAW file format, developed with linear gamma, and converted into Lab colour space. After tonal inversion of the image, luminance values of the 2706G and 7028C bands were determined with Adobe Photoshop CS4 (Adobe Systems Incorporated, San Jose, CA) using the pipette tool (11 by 11 pixel average). The background luminance values were subtracted.

## Supporting Information

Figure S1 Raw and normalized melting curves and negative 1st derivative melt-peaks obtained in the reproducibility study. Data shown were collected in two independent experiments (n = 18 real replicates per target and experiment, 32-cycle ARMS-DCA; 7500 Fast Real Time PCR System).
(2.34 MB TIF)

Figure S2 Normalized 7028C and 2706G melt-ramp heights depend on the hg H/non-hg H mixture ratio and the template molecule input. Data shown were taken from two experiments (32-cycle ARMS-DCA; 7500 Fast Real Time PCR System). Each mixture ratio/target molecule input amount combination was tested as a singleton.
(3.29 MB TIF)

Figure S3 A test-panel of nonprobative forensically relevant samples comprising reference samples (A) as well as simulated single and multi contributor crime scene stains (B-F) was analyzed with 32-cycle ARMS-DCA on a 7500 Fast Real Time PCR System. All samples were run as singletons.
(3.12 MB TIF)
