# Supplementary figures and images for: Fluorescent Duplex Allele-Specific PCR and Amplicon Melting for Rapid Homogeneous mtDNA Haplogroup H Screening and Sensitive Mixture Detection
Source: PLoS One. 2009 Dec 18;4(12):e8374. doi: 10.1371/journal.pone.0008374 (PMC2793010; doi:10.1371/journal.pone.0008374)

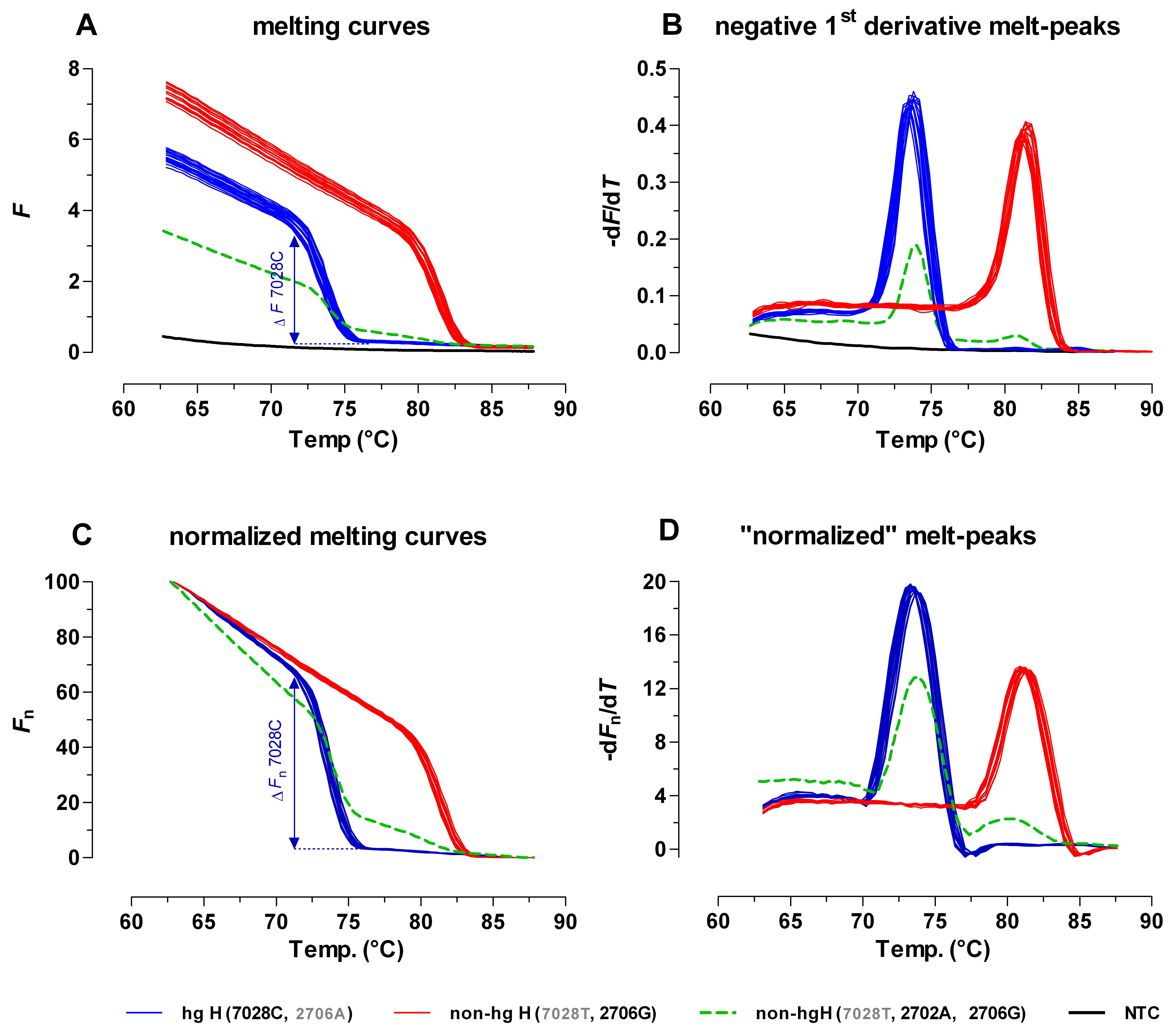

Supplement: Figure S1 — Raw and normalized melting curves and negative 1st derivative melt-peaks obtained in the reproducibility study. Data shown were collected in two independent experiments (n = 18 real replicates per target and experiment, 32-cycle ARMS-DCA; 7500 Fast Real Time PCR System). (2.34 MB TIF) [file pone.0008374.s001.tif]
